# Investigation on combined copy number variation sequencing and cytogenetic karyotyping for prenatal diagnosis

**DOI:** 10.1186/s12884-021-03918-y

**Published:** 2021-07-08

**Authors:** Jinman Zhang, Xinhua Tang, Jilin Hu, Guilin He, Jian Wang, Yingting Zhu, Baosheng Zhu

**Affiliations:** 1 Faculty of Environmental Science and Engineering, Kunming University of Science and Technology, Kunming, Yunnan 650500 People’s Republic of China; 2 Department of Obstetrics and Gynecology, First People’s Hospital of Yunnan Province, No. 157, Jinbi Road, Xishan District, Kunming, Yunnan 650032 People’s Republic of China; 3 Shanghai Children’s Medical Center, Shanghai, 200127 People’s Republic of China; 4 Research and Development Department, TissueTech, Inc., 7235 Corporate Center Drive, Suite B, Miami, Florida, 33126 USA

**Keywords:** High-throughput sequencing, copy number variation, Prenatal diagnosis, Noninvasive prenatal testing, Chromosomal diseases

## Abstract

**Background:**

We aimed to evaluate the clinical value of copy number variation-sequencing (CNV-Seq) in combination with cytogenetic karyotyping in prenatal diagnosis.

**Methods:**

CNV-Seq and cytogenetic karyotyping were performed in parallel for 9452 prenatal samples for comparison of the diagnostic performance of the two methods, and to evaluate the screening performance of maternal age, maternal serum screening, fetal ultrasound scanning and noninvasive prenatal testing (NIPT) for fetal pathogenic copy number variation (CNV).

**Results:**

Among the 9452 prenatal samples, traditional karyotyping detected 704 cases (7.5%) of abnormal cytogenetic karyotypes, 171 (1.8%) chromosome polymorphism, 20 (0.2%) subtle structural variations, 74 (0.7%) mutual translocation (possibly balanced), 52 (0.6%) without karyotyping results, and 8431 (89.2%) normal cytogenetic karyotypes. Among the 8705 cases with normal karyotype, polymorphism, mutual translocation, or marker chromosome, CNV-Seq detected 63 cases (0.7%) of pathogenic chromosome microdeletion/duplication. Retrospectively, noninvasive prenatal testing (NIPT) had high sensitivity and specificity for the screening of fetal pathogenic CNV, and NIPT combining with maternal age, maternal serum screening or fetal ultrasound scanning, which improved the screening performance.

**Conclusion:**

The combined application of cytogenetic karyotyping and CNV-Seq significantly improved the detection rate of fetal pathogenic chromosome microdeletion/duplication. NIPT was recommended for the screening of pathogenic chromosome microdeletion/duplication, and NIPT combining with other screening methods further improved the screening performance for pathogenic fetal CNV.

## Background

Different levels and types of genetic variation exist in the human genome, ranging from single nucleotide mutations to structural or numerical chromosome abnormalities. One or more genetic variations may exist in an individual, and some genetic variations cause severe congenital malformations or death. In addition to triploid and numerical chromosome abnormalities, pathogenic chromosome microdeletion/duplication also leads to poor fetal prognosis. For example, Wolf-Hirschhorn syndrome mostly results in developmental retardation, unusual faces and structural abnormalities, and Miller-Dieker syndrome can be complicated by pachygyria; 22q11.21 microdeletion syndrome often has various degrees of cardiac malformations [1–6]. If pathogenic chromosome microdeletion/duplication can be diagnosed prenatally, the births of children with such severe congenital defects can be avoided.

However, the target diseases of traditional maternal serum screening are limited to common aneuploidies. Fetal ultrasound scanning is mainly used to monitor fetal growth and development, and to find structural fetal abnormalities and soft markers. Noninvasive prenatal testing (NIPT) using maternal plasma cell-free fetal DNA has made prenatal screening for pathogenic chromosome microdeletion/ duplication possible.

Traditional cytogenetic karyotyping has been used as the gold standard diagnosis of chromosome abnormalities for decades. However, it is time-consuming and labor-intensive, largely dependent on cell culture, and has a low chromosome resolution of 5 ~ 10 Mb. In recent years, the application of high-resolution chromosome micro-array analysis (CMA), which can detect abnormal chromosome number, micro-deletions/duplication, uniparental disomy, has revolutionized the testing methodology of prenatal diagnosis. It has been suggested in some studies that CMA can be solely used instead of cytogenetic karyotyping in prenatal diagnosis laboratories with limited human resources [7]. Next-generation sequencing (NGS) now offers an alternative methodology to CMA, named copy number variation sequencing (CNV-Seq) with a resolution of 0.2 Mb for the detection of clinically significant chromosomal abnormalities. CNV-Seq has uniform sequencing coverage and relatively low price and has been gradually used in prenatal diagnosis [8]. However, more studies are required to further verify the efficiency of CNV-Seq in prenatal diagnosis.

Traditional karyotyping has characteristics of low-cost and covering the whole genome, including abnormal chromosome number and structural variation of specific regions, such as euchromatic and heterochromosomal regions. It highly depends on the experience of technicians to recognize these regions using different banding techniques under the microscope, which provide information about the frequency and location of these variations. Due to the morphologic similarity between chromosomes, karyotyping is difficult to accurately distinguish subtle structural variations. Genome copy number variation (CNVs) refers to structural variations of DNA sequence of more than 0.2 Mb. Karyotyping by conventional chromosome banding technology cannot distinguish these subtle variations. Compared with karyotyping, CNV-Seq which based on next-generation sequencing technology and comparative genomics has high resolution, high throughput, and simple laboratory operations. However, CNV-Seq also has limitations such as short read lengths and not covering the whole genome, and it cannot detect balanced translocations, polymorphism, marker chromosomes, and other genetic variations out of the detection range, and it cannot accurately detect polyploidy and low-proportion chromosome mosaic. Karyotyping and CNV-Seq are two different technologies, and the combination of them in prenatal diagnosis may make up for each other’s shortcomings and verifie each other’s results to improve the accuracy of prenatal diagnosis. Therefore, in this study, we comparatively analyzed the difference between cytogenetic karyotyping and CNV-Seq for the same fetal samples, evaluated the value of adding CNV-Seq in traditional prenatal diagnosis, analyzed the performances of maternal age, maternal serum screening, NIPT and fetal ultrasound scanning for the screening of pathogenic fetal CNV and investigated whether combined application of these prenatal screening methods could improve the sensitivity and specificity for fetal pathogenic CNV.

## Methods

### Study patients

All methods were carried out in accordance with relevant guidelines and regulations. The proposal of this study had been approved by the Ethics Committee of Institutional Research Board (IRB), First People’s Hospital of Yunnan Province before this study was conducted. The study patients were 9452 singleton pregnant women who received invasive prenatal diagnosis after informed consent forms were signed, including allowance of data management and consent for manuscript publication, in the First People’s Hospital of Yunnan Province, China, from January 2018 to December 2019. Among the study patients, 3582 (37.9%) women were served by our hospital for their prenatal care, and 5870 (62.1%) were transferred from the other hospitals because we are the provincial prenatal diagnosis center. The maternal age calculated by the expected date of confinement was 31 (27–36) years. The gestational age at prenatal diagnosis was 20 (19–21) weeks. Invasive prenatal diagnosis consisted of 8855 amniocentesis (93.7%), 552 cordocentesis (5.8%) and 44 chorion villus sampling (0.5%).

### Prenatal screening

Four types of prenatal screenings were involved in this study: (1) combined screening in 11 ~ 13^+ 6^ weeks that comprised of fetal NT measurement + maternal serum screening using pregnancy associated plasma protein-A (PAPP-A), placental growth factor (PLGF) and free human chorionic gonadotropin beta unit (fβ-HCG), with or without NIPT; (2) maternal serum screening in 16 ~ 20^+ 6^ weeks using alpha fetoprotein (AFP), fβ-HCG and unconjugated estriol (uE_3_), with or without NIPT; (3) NIPT only if gestational weeks at screening ≥21 weeks, and (4) all study patients had fetal ultrasound scanning in our department. The cases with high risk of Down Syndrome (DS), high risk of Edwards Syndrome (ES), or high risk of both by maternal serum screening were all classified as high-risk cases. Fetal ultrasound scanning was classified into five grades based on the severity of abnormalities: grade 0: without abnormal findings; grade 1: fetuses with subtly ultrasound abnormalities other than grade 2, e.g. gallbladder was not detected; grade 2: soft markers that were closely associated with chromosome aneuploidy, such as thickened nuchal fold (NF), nuchal translucency (NT) ≥ 3.0 mm, absence and/or dysplasia of nasal bone, mild to moderate ventriculomegaly, aberrant subclavian arteries, fetal growth restriction (FGR), short limb bones length, micrognathia, and acromphalus; grade 3: mild to moderate structural fetal malformations; grade 4: severe structural fetal malformations or lethal abnormalities. Maternal age ≥ 35 years at the expected date of confinement was defined as advanced maternal age.

### Invasive prenatal diagnosis

Three types of surgeries for invasive prenatal diagnosis were used in this study. Amniocentesis: Twenty mL of amniotic fluid was collected by aspiration for cell culture and cytogenetic karyotyping, and 5 mL for CNV-Seq. If amniotic fluid was contaminated by maternal blood, adherent amniocytes after cell culture were used for CNV-Seq. Cordocentesis: Five mL of amniotic fluid was collected at first for CNV-Seq, and then 1.5 mL of cord blood for cell culture and cytogenetic karyotyping, and 0.5 mL of cord blood for hemoglobin electrophoresis to exclude maternal blood contamination. In prenatal diagnosis, the standard method to exclude maternal blood contamination should be linkage analysis of DNA polymorphism. We had used the method of STR polymorphism linkage analysis. In recent years, we used hemoglobin electrophoresis instead because STR polymorphism linkage analysis was much more time-consuming and labor-intensive. Chorion villus sampling: a small amount of villous tissue was sampled for CNV-Seq directly. Before 2019, we were inexperienced in villus cell culture techniques. Therefore, the cases received chorion villus sampling were those with severe fetal structural abnormalities. No villus cell culture was applied for those cases, and only CNVs was provided.

### Laboratory testing

#### Cell culture and cytogenetic karyotyping

Amniotic fluid and umbilical blood samples were set up for cell culture following the standard protocols. Chromosome preparations were G-banded using trypsin-Giemsa staining for cytogenetic karyotyping after a series of standard protocols including colchicine treatment, hypotonic treatment, fixation and centrifugation. Karyotypes were diagnosed according to the international system for human cytogenetic nomenclature (ISCN, 2009) [9, 10]. The classification and abbreviations of abnormal karyotypes in this study were as follow: DS, ES, Patau syndrome (PS), super female syndrome (XXX), super male syndrome (XYY), Klinefelter syndrome (XXY), Turner syndrome (Turner), abnormal sex chromosome number mosaic (Sex A Mosaic), autosomal aneuploid mosaic (Auto A Mosaic), possibly balanced mutual translocation (Translocation), chromosome polymorphism (Polymorphism), triploid, chromosome fragment duplication/deletion, subtle structural variations such as inv. (21), inv. (4), dup (21), inv. (Y), inv. (1), inv. (5), inv. (12), inv. (8), inv. (19), inv. (Y), inv. (10), inv. (16). A total of 52 cases had only CNV-Seq results, but no karyotyping results. Among them, 44 cases who received chorion villus sampling, since our center cannot provide villus cell culture during that time; 8 cases encountered amniotic fluid cell culture failure. The maximum and minimum gestational weeks of amniotic fluid cell culture failure were 31 and 20 weeks, respectively. It should be noted that none of the 52 cases with missing results were used in the data analysis of this study.

#### CNV-Seq and result interpretation

Nextseq 550AR platform (Illumina, San Diego, CA) was used for DNA sequencing, with an average sequencing depth of 0.08×, following the Q30 sequencing quality standard. The amount of fetal DNA used for CNV-Seq was 10 ~ 50 ng for each prenatal sample. AnnoroadPD software (Annoroad Gene Technology Co., Ltd., Beijing, China) was applied to analyze the sequencing data referring to the human reference genome GRCh37/hg19. The identified fetal CNV were interpreted [11] and classified into five categories: pathogenic (P-), likely pathogenic (LP-), uncertain significance (VUS-), likely benign (LB-) and benign (B-), according to the standards and guidelines that were jointly developed by the American College of Medical Genetics and Genomics (ACMG), the Association for Molecular Pathology (AMP) and the College of American Pathologists (CAP) in 2015. To conveniently show the CNV-Seq results, we used “P-“as abbreviation for pathogenic chromosome microdeletion/duplication, “None” for no copy number variation found, “auto A” for autosomal aneuploidy, “sex A” for abnormal sex chromosomes number, “auto AM” for autosomal aneuploidy mosaic, and “sex AM” for abnormal sex chromosomes number mosaic. Cytogenetic karyotyping was the diagnostic method for numerical and structural chromosome abnormalities, and high-throughput sequencing for CNV. For LP- and VUS-, family (parents and fetuses) CNV-Seq tests, fluorescence in-situ hybridization (FISH) or multiplex ligation probe amplification (MLPA) were used for further verification.

### Statistical analysis

The data were analyzed statistically using IBM SPSS Statistics (version 22.0, IBM Corp., Armonk, NY, USA). Continuous variables (for example, age and gestational weeks at prenatal diagnosis) were expressed as “median [lower quartile, upper quartile]”, and analyzed using Kruskal-Wallis one-way analysis of variance. Categorical variables are represented by “n (%)” and analyzed using Chi-square test for two-way disordered R × C table. Calculation for sensitivity and specificity: sensitivity = true positive / (true positive + false negative) *100%; specificity = true negative / (true negative + false positive) *100%. Paired chi-square test was used to test the difference between CNV-Seq and various prenatal screening methods and prenatal diagnosis results (*P* <  0.01 was considered statistically significant). Missing items were not applied in data analysis.

## Results

### Basic characteristics of study patients

Basic information of 9452 cases of prenatal diagnosis was listed and statistically analyzed in Table 1. Among study patients, 9452 (100%) had received one to two times of fetal ultrasonography in our center, 5688 (60.2%) had maternal serum screening, 1409 (14.9%) had NIPT, 551 (0.58%) had both NIPT and maternal serum screening, and 3142 cases (33.2%) were in advanced maternal age. The results showed that only 1165 (12.3%) of patients received invasive prenatal diagnosis due to high risk of NIPT. Other indications for prenatal diagnosis included advanced maternal age, abnormal fetal ultrasound scanning, high risk of maternal serum screening, adverse reproductive history, family history of single-gene genetic diseases, or others.

**Table 1:** Characteristics of study population

Characteristic	Study population (*n* = 9452)
Maternal age (years)	31 (27–36)
Advanced maternal age (≥ 35 yrs)	3142 (33.2)
Nation	
Han	6593 (69.8)
Yi	822 (8.7)
Bai	433 (4.6)
Dai	282 (3.0)
Hui	250 (2.6)
Zhuang	167 (1.8)
Naxi	145 (1.5)
Hani	112 (1.2)
Others	648 (6.8)
Parity	
Nulliparous	3457 (36.6)
Parous	5995 (63.4)
= 1	5355 (56.7)
= 2	567 (6.0)
≥ 3	64 (0.7)
Gestational age at invasive diagnosis (weeks)	20 (19–21)
Invasive prenatal diagnosis procedure	
Amniocentesis	8855 (93.7)
Cordocentesis	552 (5.8)
Chorion villus sampling	44 (0.5)
Maternal or/and paternal chromosome abnormalities	155 (1.6)
History of bearing child with chromosome abnormalities	203 (2.1)
Indications for invasive prenatal diagnosis	
NIPT high-risk	415 (4.4)
Maternal serum screening high-risk	1984 (21.0)
DS high-risk	2999 (31.7)
ES high-risk	799 (8.5)
Both DS and ES high-risk	
Advanced maternal age (≥ 35 yrs)	1177 (12.5)
Abnormal fetal ultrasonography	1229 (13.0)
Grade 1	1857 (19.6)
Grade 2	1549 (16.4)
Grade 3	531 (5.6)
Grade 4	219 (2.3)
Other indications	831 (8.8)
≥ two indications	3816 (40.4)

### Comparison of results between CNV-Seq and cytogenetic karyotyping

The results of 9452 cases of cytogenetic karyotyping were listed in Table 2: a total of 704 (7.5%) cases of fetal chromosome abnormalities, 171 (1.8%) chromosomal polymorphism, 20 (0.2%) subtle structural variations, 74 (0.7%) mutual translocation (possibly balanced), 52 (0.6%) without karyotyping results, and 8431 (89.2%) normal karyotypes were detected.

**Table 2:** Comparison of results between cytogenetic karyotyping and CNV-Seq

cytogenetic karyotyping		CNV										*P* value
	None (*n* = 4851)	B (*n* = 38)	LB (*n* = 3465)	VUS (*n* = 304)	LP (*n* = 23)	P-del/dup (*n* = 118)	Auto A (*n* = 446)	Auto A M (*n* = 18)	Sex A (*n* = 156)	Sex A M (*n* = 33)	Total (*n* = 9452)	
Normal	4666 (96.2)	36 (94.7)	3355 (96.8)	289 (95.1)	22 (95.7)	60 (50.8)	0 (0.0)	1 (5.6)	1 (0.6)	1 (3.0)	8431 (89.2)	< 0.001
DS	0 (0.0)	0 (0.0)	0 (0.0)	0 (0.0)	0 (0.0)	0 (0.0)	358 (80.3)	0 (0.0)	0 (0.0)	0 (0.0)	358 (3.8)	
ES	0 (0.0)	0 (0.0)	0 (0.0)	0 (0.0)	0 (0.0)	0 (0.0)	55 (12.3)	0 (0.0)	0 (0.0)	0 (0.0)	55 (0.6)	
PS	0 (0.0)	0 (0.0)	0 (0.0)	0 (0.0)	0 (0.0)	0 (0.0)	12 (2.7)	0 (0.0)	0 (0.0)	0 (0.0)	12 (0.1)	
XXX	0 (0.0)	0 (0.0)	0 (0.0)	0 (0.0)	0 (0.0)	0 (0.0)	0 (0.0)	0 (0.0)	27 (17.3)	1 (3.0)	28 (0.3)	
XYY	0 (0.0)	0 (0.0)	0 (0.0)	0 (0.0)	0 (0.0)	0 (0.0)	0 (0.0)	0 (0.0)	33 (21.2)	0 (0.0)	33 (0.3)	
XXY	0 (0.0)	0 (0.0)	0 (0.0)	0 (0.0)	0 (0.0)	0 (0.0)	0 (0.0)	0 (0.0)	72 (46.2)	0 (0.0)	72 (0.8)	
mark	2 (0.0)	1 (2.6)	2 (0.1)	2 (0.7)	0 (0.0)	2 (1.7)	0 (0.0)	0 (0.0)	0 (0.0)	0 (0.0)	9 (0.1)	
Turner	0 (0.0)	0 (0.0)	0 (0.0)	0 (0.0)	0 (0.0)	0 (0.0)	0 (0.0)	0 (0.0)	11 (7.1)	2 (6.1)	13 (0.1)	
Sex A Mosaic	4 (0.1)	0 (0.0)	4 (0.1)	1 (0.3)	0 (0.0)	2 (1.7)	0 (0.0)	0 (0.0)	3 (1.9)	28 (84.8)	42 (0.4)	
Auto A Mosaic	2 (0.0)	0 (0.0)	1 (0.0)	0 (0.0)	0 (0.0)	0 (0.0)	2 (0.4)	16 (88.9)	0 (0.0)	0 (0.0)	21 (0.2)	
Translocation	47 (1.0)	0 (0.0)	23 (0.7)	3 (1.0)	0 (0.0)	1 (0.8)	0 (0.0)	0 (0.0)	0 (0.0)	0 (0.0)	74 (0.8)	
Polymorphism	102 (2.1)	0 (0.0)	63 (1.8)	5 (1.6)	0 (0.0)	0 (0.0)	0 (0.0)	1 (5.6)	0 (0.0)	0 (0.0)	171 (1.8)	
Triploid	2 (0.0)	0 (0.0)	0 (0.0)	0 (0.0)	0 (0.0)	0 (0.0)	0 (0.0)	0 (0.0)	0 (0.0)	0 (0.0)	2 (0.0)	
Unbalance	0 (0.0)	0 (0.0)	0 (0.0)	2 (0.7)	0 (0.0)	49 (41.5)	0 (0.0)	0 (0.0)	0 (0.0)	0 (0.0)	51 (0.5)	
No results #	14 (0.3)	1 (2.6)	9 (0.3)	2 (0.7)	1 (4.3)	2 (1.7)	18 (4.0)	0 (0.0)	5 (3.2)	0 (0.0)	52 (0.6)	
Sex and other	0 (0.0)	0 (0.0)	0 (0.0)	0 (0.0)	0 (0.0)	2 (1.7)	1 (0.2)	0 (0.0)	4 (2.6)	1 (3.0)	8 (0.1)	
Others	12 (0.2)	0 (0.0)	8 (0.2)	0 (0.0)	0 (0.0)	0 (0.0)	0 (0.0)	0 (0.0)	0 (0.0)	0 (0.0)	20 (0.2)	

The results of CNV-Seq in Tables 2, 8,354 fetuses with CNV-Seq findings were included as None, B-, and LB-, cytogenetic karyotyping showed that except for 2 cases of triploid, the rest 271 cases of abnormal karyotypes had good prognosis. A total of 530 cases of fetal aneuploidies (DS, ES, PS, XXY, XYY) were diagnosed, and the results of karyotyping and CNV-Seq were consistent. The details for 60 cases of pathogenic microdeletion/duplication detected by CNV-Seq were shown in Table 3. CNV-Seq detected 1 case of chromosomal aneuploidy and 2 cases of mosaic in fetuses with normal cytogenetic karyotypes. Furthermore, 2 cases (No.29 and 30) of pathogenic microdeletion/duplication were detected in 9 fetuses with marker chromosomes, and 1 case (No.38) of pathogenic microdeletion/duplication were detected in fetuses with mutual translocations (Seen in Table 4). Therefore, we may conclude that the combination of the two methodologies significantly improved the accuracy of prenatal diagnosis for fetal pathogenic CNV and was helpful to assess fetal prognosis. Due to its detection limitations, for example, two cases of triploid by karyotyping had normal CNV-Seq results, CNV-Seq could not replace karyotyping at present stage but might be an effective complement.

**Table 3:** Clinical data of 60 cases with normal karyotype but pathogenic microdeletion/duplication

Pathogenic microdeletion/duplication	(n)	Termination of pregnancy (n)	Continued pregnancy(n)	Pregnancy outcomes
Xp22.31 deletion X-linked ichthyosis	17	6	11	3 female fetuses, 2 of which continued pregnancy, and 1 of which terminated due to severe type thalassaemia. 14 male fetuses, 9 of which continued pregnancy, and 5 of which terminated.
22q11.21 deletion Digeorge syndrome	7	6	1	The pregnant women have mild mental retardation, fetus had right aortic arch, maternal derived 22q11.21 microdeletion, the couple chose to continue pregnancy
Xp21.1 deletion Duchenne muscular dystrophy	1	1	0	Male fetus, the couple chose to terminate the pregnancy。
Xp21.1 duplication Duchenne muscular dystrophy	1	0	1	Female fetus, the couple chose to continue the pregnancy。
Other pathogenic autosomal microdeletion/duplication	34	30	4	2 cases of 17p12 deletion (1 case was maternal origin), 1 case of 1q21.1-q21.2 deletion (maternal origin), 22q11.2 microduplication (paternal origin), the couple chose to continue the pregnancy.
Total	60	44	16

**Table 4:** Forty five cases with inconsistent karyotyping and CNV-seq results

No.	Indications for prenatal diagnosis	Abnormal karyotypes	CNV-seq results	Fetal ultrasound	Pregnancy outcomes
1	Advanced maternal age	69,XXX	46,XX	Fetal growth restrction, tethered spinal cord, ankle joint reflexion, diaphragm expansion	Termination of pregnancy
2	Maternal serum screening ES high risk、NIPT low-risk	69,XXX	46,XX	Fetal trunk is significantly smaller than the head, left lung absent, double Outlet Right Ventricle	Termination of pregnancy
3	NIPT high-risk	47,XXX	46XX[20%]/47XXX[80%]	Normal	Gave birth to a girl
4	NIPT high-risk	45,X[18]/46,X,i(X)(p10)[34]	45,X,del(Xp11.21-p22.33)52.45mb(73%)/46,XX(27%)	Thickened nuchal folder	Continue pregnancy
5	NIPT high-risk	45, X	46,XX[11%]/45,X[89%]	Fetal growth restrction	Termination of pregnancy
6	NIPT high-risk	45,X[53]/47,XXX [5]	45,X	Ventricular septal defect	Termination of pregnancy
7	Advanced maternal age	45,X[18]/46,X,+mar[24]	45X with possible X structure abnormality	Mild bilateral renal hydrops, bilateral ventriculomegaly, slightly larger right heart	Termination of pregnancy
8	NIPT high-risk	45,X[19]/46,XY [16]	Turner mosaic	Bilateral renal pelvis separation	Termination of pregnancy
9	NIPT high-risk	45,X[35]/46,XX [9]	45,X	Normal	Termination of pregnancy
10	Maternal serum screening high risk	47,XYY [7]/46, XY[56]	Y chromosome duplication(16.85 Mb)	Normal	Gave birth to a boy
11	NIPT high-risk	45,X[22]/46,XY [8]	Yq11.221-q11.223 deletion(VUS)	Bowel echo enhancement	Termination of pregnancy
12	Maternal serum screening high risk	45,X[37]/46,XY [7]	4p15.33、Yq11.222-q11.223 deletion(VUS)	Normal	Termination of pregnancy
13	Childbearing history of gastrodialysis	45,X [10]/46,XX[42]	Likely benign variation	Left nasal bone dysplasia, right Nasal bone absent	Gave birth to a girl
14	NIPT high-risk	45X [5]/46XX	Likely benign variation	Retract chin and lower lip	Continue pregnancy
15	NIPT high-risk	45,X [8]/46,XX[92]	Likely benign variation	Normal	Termination of pregnancy
16	NIPT high-risk	45,X [4]/46,XX[51]	Likely benign variation	Normal	Continue pregnancy
17	Couples are thalassaemia carrier	45,X [10]/46,XY[28]	Normal	Normal	Termination of pregnancy
18	Advanced maternal age	47,XXY[10]46,XY[45]	Normal	Bilateral renal pelvis separation, bowel echo enhancement	Gave birth to a boy
19	NIPT high-risk	45,X[34]/47,XXX[26]	Normal	Bilateral renal pelvis separation	Termination of pregnancy
20	Couples are thalassaemia carrier	45,X [5]/46,XY[40]	Normal	The fetus is smaller 8 days than gestational week	Gave birth to a boy
21	NIPT high-risk	47,XN,+ 21[18]/46,XN[31]	47,XN,+ 21[58%]	Small humerus and femoral length, small head circumference	Termination of pregnancy
23	NIPT high-risk	47,XX,+ 21[27]/46,XX [8]	DS	Normal	Termination of pregnancy
24	NIPT high-risk	47,XX,+ 18[29] /46,XX [5](GTG)	47,XN,+ 18[78%]/46,XN[22%]	Incontinuity of lower part of cerebellar vermis, complete endocardial cushion defect	Termination of pregnancy
25	NIPT high-risk	47,XN,+ 15[4]/46,XN[51]	Trisomy 15 mosaic (50%)	Single umbilical artery	Termination of pregnancy
26	NIPT high-risk	47,XY,+ 5[15]/46,XY[47]	CNVs benign variation	FGR, ventricular septal defect, thickened right ventricular wall, Aorta straddle, enhanced echo of the aortic valve, tricuspid valve and intestinal echo	Termination of pregnancy
27	NIPT high-risk	47,XY,+ 13 [5]/46,XY [63]	CNVs-	Normal	Gave birth to a healthy boy
28	Advanced maternal age	47,XY,+ 18 [5]/46,XY [90]	CNVs(−)	Polyhydramnios	Gave birth to a healthy boy
29	NIPT high-risk	47,XN,+mar	12p12.1-p13.33 and 21q11.2-q22.11duplication(pathogenic)	Normal	Termination of pregnancy
30	Maternal serum screening high risk	mos46,X,+mar[23]/45,X [14]	Xp11.21-p22.33 deletion 56.8mb and Xq21.31-q28 deletion 64.6mb(pathogenic)	Short humerus and femoral length, Ventricular Septal Defect	Termination of pregnancy
31	Advanced maternal age	47,XN,+mar	2q11.1-q11.2 duplication(VUS)	Bilateral choroid plexus cysts, enhanced bowel echo	Lost to follow-up
32	Advanced maternal age	47,XX,+mar	5q21.2-q21.3 duplication, VUS	Normal	Continue pregnancy
33	Amniotic fluid 46,XN[38] /47,XN,+mar[22]	Cord blood 47,XX,+mar [17]/46,XX [17]	dup(8q24.22)Likely benign variation	Normal	Continue pregnancy
34	NIPT: abnormal chromosome 3 number	47,XX,+mar	Likely benign variation	Short nasal bone	Gave birth to a healthy girl
35	Fetal acromphalus	47,XY,+mar [11]/46,XY[31]	Likely benign variation	Acromphalus, edema	Termination of pregnancy
36	Thalassaemia?	47,XX,+mar [13]/46,XX[62]	Likely benign variation	Normal	Gave birth to a healthy girl
37	Advanced maternal age	47,XY,+mar [7]/46,XY[33]	Normal	Normal	Gave birth to a healthy boy
38	NIPT high-risk	46,XN,t(1;13)(q25;?q22)de novo	13q14.3-q21.33 deletion 23.1mb(pathogenic)	Normal	Termination of pregnancy
39	Childbearing history of deaf children	46,XY,dup(1)(q21.2)?	1q521.2 duplication(VUS)	Normal	Gave birth to a healthy boy
40	NIPT high-risk	46,X,del(Y)(q11)?	Xp22.31-p22.33 duplication VUS	Fetal right ventricular wall has strong echo and was thickened	Lost to follow-up
41	NIPT high-risk	46,XN,inv.(9)(p12q13)[79]	DS mosaic[20%]	Normal	Lost to follow-up
42	Maternal serum screening high risk, Advanced maternal age	45,X,der(13)t(Y;13)(q11.2?;p10?)[26]/45,X [5]	X,del(Y)[75%]/XO[25%]	FGR?	Termination of pregnancy
43	Maternal serum screening high risk	46,XY[45]	XY[60%]/XYY[40%]	Right aortic arch	Continue pregnancy
44	NIPT high-risk	46,XX[40]	XXY Gene detection:SRY existed, AZF all missing	Male genitalia	Gave birth to a healthy boy, 2 years-old
45	NIPT high-risk	46,XY[83]	47,XN,+ 2[23%]/46,XN[77%]	Normal	Termination of pregnancy

### Performances of maternal age, maternal serum screening, NIPT and fetal ultrasound scanning for pathogenic CNV-Seq results and pathogenic karyotypes

The target diseases of maternal serum screening are common chromosomal aneuploidies. For fetuses with pathogenic microdeletion/duplication, the prognosis is mostly poor. NIPT is the ideal prenatal screening method for pathogenic microdeletion/duplication, but the cost may limit its clinical use to a certain extent. If NIPT was unavailable, we wondered whether other screening methods could recognize pathogenic microdeletion/duplication. In this study, we retrospectively analyzed the results of maternal age, maternal serum screening, NIPT, and fetal ultrasound scanning for women with pathogenic fetal CNV-Seq results, including pathogenic microdeletion/duplication and Auto A, Sex A, Auto AM, Sex AM, as shown in Table 5**.** NIPT missed one case of Auto AM (CNV-Seq) and Triploid (karyotypes), shown in Table 6. In maternal serum screening, 55.2% of pathogenic microdeletion/duplication (CNV-Seq) and 42.1% of unbalanced fragment deletion/duplication (karyotypes) showed high risks results. As to abnormal ultrasound findings (grades 2–4), there was significant difference between CNV-Seq of auto A and pathogenic microdeletion/ duplication (73.6% versus 43.2%, *P* <  0.001). Regarding to pathogenic karyotypes, NIPT missed one case of triploid, whose maternal serum screening and fetal ultrasound were abnormal. Only 42.2% of severe chromosomal abnormalities (aneuploidy, unbalanced fragment deletion/duplication, triploidy) were screened out by advanced maternal age. The detection rate of abnormal fetal ultrasound findings (grade 2–4) in fetuses with abnormal karyotypes were DS 69.9%, ES 85.5%, PS 91.7%, unbalanced fragment deletion/duplication 56.8%, triploid 100%, XXX 14.3%, XYY 24.2%, and XXY 18.1%. We speculated that there might be a dose-effect between fetal ultrasound abnormalities and chromosomal diseases, and a difference between autosomal and sex chromosomal abnormalities. Therefore, maternal age, maternal serum screening, fetal ultrasound scanning and NIPT all had certain predictive values for pathogenic CNV-Seq results and pathogenic karyotypes (chromosomal aneuploidy, unbalanced segment deletion/duplication, and triploid).

**Table 5:** Performances of karyotyping over CNV-seq in each indication for prenatal diagnosis

Indication for prenatal diagnosis	P-del/dup (*n* = 118)		Auto A (*n* = 446)		Auto A M (*n* = 18)		Sex A (*n* = 156)		Sex A M (*n* = 33)	Total (*n* = 771)	*P* value
NIPT											
NIPT High-risk	30 (25.40)		272 (61.00)		11 (61.00)		125 (80.10)		21 (63.60)	459 (59.53)	< 0.001
NIPT Low-risk	0 (0.00)		0 (0.00)		1 (6.00)		0 (0.00)		0 (0.00)	1 (0.13)	
Absent	88 (74.60)		174 (39.00)		6 (33.00)		31 (19.90)		12 (36.40)	311 (40.34)	
Maternal serum screening											
Maternal serum screening High-risk	32 (27.12)		129 (28.90)		6 (33.33)		8 (5.10)		13 (39.40)	188 (24.40)	< 0.001
Maternal serum screening Low-risk	26 (22.03)		42 (9.40)		4 (22.22)		33 (21.20)		6 (18.20)	111 (14.40)	
Absent	60 (50.85)		275 (61.70)		8 (44.44)		115 (73.70)		14 (42.40)	472 (61.20)	
fetal ultrasound											
fetal ultrasound (0)	47 (39.83)		82 (18.00)		9 (50.00)		88 (56.41)		13 (39.40)	239 (31.00)	< 0.001
fetal ultrasound (1)	20 (16.95)		36 (8.00)		5 (28.00)		27 (17.31)		12 (36.36)	100 (13.00)	
fetal ultrasound (2)	15 (12.71)		177 (40.00)		1 (5.50)		18 (11.54)		5 (15.15)	216 (28.00)	
fetal ultrasound (3)	20 (16.95)		78 (18.00)		2 (11.00)		13 (8.33)		2 (6.06)	115 (14.90)	
fetal ultrasound (4)	16 (13.56)		73 (16.00)		1 (5.50)		10 (6.41)		1 (3.03)	101 (13.10)	
maternal age											
Advanced maternal age	22 (18.60)		210 (47.10)		7 (38.90)		49 (31.40)		12 (36.40)	300 (38.90)	< 0.001
maternal age < 35 years old	96 (81.40)		236 (52.90)		11 (61.10)		107 (68.60)		21 (63.60)	471 (61.10)	
Indication for prenatal diagnosis	DS (*n* = 358)	ES (*n* = 55)	PS (*n* = 12)	XXX (*n* = 28)	XYY (n = 33)	XXY (*n* = 72)	XO (*n* = 13)	Triploid (*n* = 2)	Unbalance (*n* = 51)	Total (*n* = 624)	*P* value
NIPT											
NIPT High-risk	231 (64.50)	30 (54.50)	7 (58.30)	22 (78.60)	29 (87.90)	65 (90.30)	7 (53.80)	0 (0.00)	16 (31.40)	407 (65.20)	< 0.001
NIPT Low-risk	0 (0.00)	0 (0.00)	0 (0.00)	0 (0.00)	0 (0.00)	0 (0.00)	0 (0.00)	1 (50.00)	0 (0.00)	1 (0.20)	
Absent	127 (35.50)	25 (45.50)	5 (41.70)	6 (21.40)	4 (12.10)	7 (9.70)	6 (46.20)	1 (50.00)	35 (68.60)	216 (34.60)	
Maternal serum screening											
Maternal serum screening High-risk	99 (27.65)	23 (41.80)	4 (33.30)	0 (0.00)	2 (6.06)	3 (4.20)	3 (23.10)	1 (50.00)	8 (15.70)	143 (22.90)	< 0.001
Maternal serum screening Low-risk	39 (10.90)	0 (0.00)	2 (16.70)	8 (28.60)	6 (18.18)	17 (23.60)	1 (7.70)	0 (0.00)	11 (21.60)	84 (13.50)	
Absent	220 (61.45)	32 (58.20)	6 (50.00)	20 (71.40)	25 (75.76)	52 (72.20)	9 (69.20)	1 (50.00)	32 (62.70)	397 (63.60)	
Fetal ultrasound											
fetal ultrasound (0)	77 (21.51)	3 (5.45)	1 (8.30)	18 (64.30)	21 (64.00)	46 (64.00)	1 (8.00)	0 (0.00)	17 (33.33)	184 (29.00)	< 0.001
fetal ultrasound (1)	31 (8.66)	5 (9.09)	0 (0.00)	6 (21.40)	4 (12.00)	13 (18.00)	2 (15.00)	0 (0.00)	5 (9.80)	66 (11.00)	
fetal ultrasound (2)	169 (47.21)	4 (7.27)	0 (0.00)	1 (3.60)	4 (12.00)	10 (14.00)	5 (38.00)	0 (0.00)	9 (17.65)	202 (32.00)	
fetal ultrasound (3)	55 (15.36)	16 (29.09)	2 (16.70)	2 (7.10)	4 (12.00)	2 (3.00)	1 (8.00)	0 (0.00)	11 (21.57)	93 (15.00)	
fetal ultrasound (4)	26 (7.26)	27 (49.10)	9 (75.00)	1 (3.60)	0 (0.00)	1 (1.00)	4 (31.00)	2 (100.00)	9 (17.65)	79 (13.00)	
Maternal age											
Advanced maternal age	179 (50.00)	23 (41.80)	3 (25.00)	14 (50.00)	4 (12.10)	27 (37.50)	0 (0.00)	1 (50.00)	7 (13.70)	258 (41.30)	< 0.001
unadvanced maternal age	179 (50.00)	32 (58.20)	9 (75.00)	14 (50.00)	29 (87.90)	45 (62.50)	13 (100.00)	1 (50.00)	44 (86.30)	366 (58.70)	

*P* values were the statistical difference of constituent ratios by Chi-square test between NIPT and CNV-seq, maternal serum screening and CNV-seq, fetal ultrasound and CNV-seq, maternal age and CNV-seq

**Table 6:** Cases with high risk of combined screening test but low risk NIPT

	Maternal serum screening	Fetal ultrasound	Maternal age (years)	Fetal CNV-seq results	Fetal karyotypes	Pregnancy outcomes
1	N/A	Ventriculomegaly, cardiac malformations, pulmonary dysplasia	28	47,XN,+ 13[40%]/46,XN[60%]	47,XY,+ 13[12]/46,XY[74]	Termination of pregnancy
2	High risk	Imbalance of head-body ratio, double outlet right ventricle, absent left lung	27	Normal	69,XXX	Termination of pregnancy
3	Low risk	Nasal bone dysplasia	30	Likely benign variation	46,XX,t(11;17)(q21;q23)	Continue pregnancy
4	N/A	Right aortic arch	36	Likely benign variation	46,XN,inv.(9)(p12q13)	Continue pregnancy
5	Low risk	Enhanced echo in both kidneys and intestine, pleural effusion	31	Likely benign variation	46,XX,t(12;22)(q24.1;q13)	Continue pregnancy
6	Threshold risk	Right choroid plexus cyst	28	Likely benign variation	46,XY,1qh+	Continue pregnancy
7	Low risk	Normal	40	Normal	46,XX,inv.(19)(p13.3q13.1)	Continue pregnancy
8	N/A	Normal	36	Normal	46,XY,t(8;16)(q12;q21)mat	Continue pregnancy
9	N/A	Duodenal atresia	23	Normal	46,XX,t(2;7)(q13;q22)	Continue pregnancy
10	N/A	Nasal bone absent	38	Normal	46,XY,15 ps+	Continue pregnancy
11	N/A	Thickened ventricular wall, small heart size, enlarged liver and spleen	26	Normal	46,XY,21 ps+	
12	Low risk	Holoprosencephaly, clearly displayed nasal bones, incontinuity of upper alveolar process	31	Normal	Chorionic villus sampling, no karyotyping	Termination of pregnancy
13	N/A	Ventricular septal defect, hemivertebra, scoliosis	26	Normal	Normal	Termination of pregnancy
14	N/A	Holoprosencephaly, agenesis of corpus callosum, hydrocephalus	33	Normal	Normal	Termination of pregnancy
15	N/A	Absent right lung, Tetralogy of Fallot, hemivertebrae	25	Normal	Normal	Termination of pregnancy
16	Threshold risk	Complete endocardial cushion defect	25	Normal	Normal	Termination of pregnancy
17	N/A	Left ventricular rhabdomyomas, strephenopodia	36	Normal	Normal	Termination of pregnancy
18	Low risk	Left microtia, nasal dysplasia, atresia of nostril	35	Normal	Normal	Termination of pregnancy
19	N/A	Ventricular septal defect, pulmonary artery stenosis, missing pubic bones, hooked hands	33	Normal	Normal	Termination of pregnancy

As seen in Table 7, for pathogenic fetal CNV-Seq results, NIPT had the highest sensitivity of 1.00 (0.99–1.00) but lowest specificity of 0.22 (0.18–0.26), while maternal serum screening had higher sensitivity of 0.63 (0.57–0.68) and lower specificity of 0.37 (0.35–0.39). The sensitivity and specificity of fetal ultrasound scanning were 0.69 (0.66–0.72) and 0.59 (0.57–0.60), respectively. Advanced maternal age had a specificity of 0.68 (0.67–0.70) and a sensitivity of 0.39 (0.35–0.42).

**Table 7:** Predictive efficiencies of single indication for pathogenic fetal CNV-seq results

		CNV		Total	McNemar’s χ 2	*P* value	Sensitivity	Specificity
		1	0					
NIPT	1	459	339	798	334.03	< 0.001	1.00 (0.99, 1.00)	0.22 (0.18, 0.26)
	0	1	96	97				
Total		460	435	895				
Maternal serum screening	1	188	1517	1705	1212.5	< 0.001	0.63 (0.57, 0.68)	0.37 (0.35, 0.39)
	0	111	884	995				
Total		299	2401	2700				
fetal ultrasound	1	532	1585	2117	991.79	< 0.001	0.69 (0.66, 0.72)	0.59 (0.57, 0.60)
	0	239	2245	2484				
Total		771	3830	4601				
Advanced maternal age	1	300	1207	1507	321.95	< 0.001	0.39 (0.35, 0.42)	0.68 (0.67, 0.70)
	0	471	2623	3094				
Total		771	3830	4601				

CNV

1-including pathogenic microdeletion/duplication, Auto A, Sex A, Auto AM, Sex AM

0-including likely pathogenic (LP-), uncertain significance (VUS-), likely benign (LB-) and benign (B-)

NIPT.

1-high risk.

0-low risk.

Maternal serum screening.

1-high risk.

0-low risk.

Fetal ultrasound.

1-abnormal ultrasound findings (grades 2–4).

0-normal or subnormal ultrasound findings (grades 0–1).

Advanced maternal age

1-Advanced maternal age (≥ 35 yrs)

0-Maternal age (< 35 yrs).

### Correlation between fetal CNV-Seq and indications for prenatal diagnosis

According to the number of indications for prenatal diagnosis, the 9452 women were divided into four groups: (1) single indication, (2) positivity for any two or (3) three or (4) four indications. Indications included high risk in NIPT, high risk in maternal serum screening, fetal ultrasound abnormalities (Grade 2–4), advanced maternal age, and other indications such as adverse childbearing history except monogenic diseases. The sensitivity and specificity of different prenatal diagnostic indications for the screening of pathogenic microdeletion/microduplication were lists in Table 8. Single indication had the highest sensitivity and the lowest specificity. In a similar trend, four indicators had the lowest sensitivity and the highest specificity. Accordingly, to achieve the optimal sensitivity and specificity, a prenatal screening program that combines two methods could be considered.

**Table 8:** Sensitivity and Specificity of single or multiple indications for fetal pathogenic CNV

		CNV		Total	McNemar’s χ 2	*P* value	Sensitivity	Specificity
		1	0					
Positive for single indication	1	746	3315	4061	3286.1	< 0.001	0.99 (0.98, 0.99)	0.10 (0.09, 0.11)
	0	9	356	365				
Total		755	3671	4426				
Positive for two indications	1	746	3315	4061	546.15	< 0.001	0.69 (0.65, 0.72)	0.70 (0.69, 0.72)
	0	9	356	365				
Total		755	3671	4426				
Positive for three indications	1	180	88	268	356.25	< 0.001	0.24 (0.21, 0.27)	0.98 (0.97, 0.98)
	0	575	3583	4158				
Total		755	3671	4426				
Positive for four indications	1	15	1	16	751.01	< 0.001	0.02 (0.01, 0.03)	1.00 (1.00, 1.00)
	0	740	3670	4410				
Total		755	3671	4426				

## Discussion

### Combination of cytogenetic karyotyping and CNV-Seq can prenatally diagnose more fetal pathogenic microdeletion/duplication and provide comprehensive prenatal information

In addition to traditional cytogenetic karyotyping, CMA and CNV-Seq have gradually been used in prenatal diagnosis. CNV-Seq is worth applying in prenatal diagnosis due to its lower cost and uniform sequencing coverage. Fetuses with normal karyotype/chromosome polymorphism usually have good prognosis, but pathogenic microdeletion/duplication cannot be excluded. In this study, among fetuses with normal karyotypes, CNV-Seq diagnosed 60 (0.6%) cases of pathogenic CNV, and fortunately the births of 44 fetuses with poor prognosis were avoided. A complex case was also prenatal diagnosed with CNV-Seq result of 47,XXY and karyotyping result of 46,XX. SRY gene detection indicated a male gender, but all 6 loci on AZF gene were missing. Fetal ultrasound showed male external genitalia. Taking fetal ultrasound findings together, the fetus might have sexual reversal and risks of abnormal reproductive system development in puberty. The parents were fully informed the advantages, disadvantages and limitations of karyotyping and CNV-Seq, and to make clear that the two results cannot be denied by each other but be mutually complementary. The couple chose to continue the pregnancy. The boy is now 1.5 years old and is generally healthy. Follow-up and etiological examination were recommended. On the other hand, we should make clear that not all fetuses with structural chromosomal abnormalities by karyotyping have poor prognosis. For example, mutual translocation (paternal/maternal/de novo) with normal CNV-Seq and fetal ultrasound is very much likely that the fetuses have good prognosis. The combination of karyotyping and CNV-Seq enables mutual verification of the results in prenatal diagnosis and helps to avoid misdiagnosis and provide more information for comprehensive evaluation of fetal prognosis. However, the combined application of CNV-Seq and karyotyping may lead to increased economic burden. It needs further verification about whether the cost-effectiveness is worth promoting. We need to choose an appropriate prenatal diagnosis program based on our own characteristics.

### Combined several prenatal screening significantly improves the specificity but reduces the sensitivity for fetal pathogenic CNV

Maternal serum screening detects 70 ~ 80% of DS, at a false positive rate of 5% [12]. It was reported that a small portion of sex chromosome abnormalities showed abnormal findings in maternal serum screening [13]. It is unknown whether maternal serum screening is abnormal for pathogenic CNV. The findings of this study indicated that maternal serum screening can detect 55.2% of fetal pathogenic chromosomal microdeletion/ duplication in fetuses with normal or abnormal karyotype. The intrauterine phenotype of fetuses with pathogenic microdeletion/ duplication lacks specificity, so prenatal ultrasound scanning is difficult to identify. In this study, fetuses with autosomal aneuploidy had the most severe ultrasound abnormalities, followed by pathogenic microdeletion/duplication with a large variability that some fetuses had completely normal ultrasonography. Fetuses with abnormal sex chromosome number and structure and the mosaic had mild ultrasound abnormalities. Fetal ultrasound scanning had higher sensitivity for autosomal aneuploidy. However, if other prenatal screening methods such as NIPT and maternal serum screening are unavailable at the same time, the ultrasonologist’s subjective assessments of fetal subtle facial features/minor heart variations are inadequate for accurate assessment of fetal prognosis, and the clinical value is limited. NIPT has been widely used as a first-line prenatal screening method [14]. It was reported that the sensitivities of NIPT for Trisomy 21, 18, 13 are 99.1, 98.2 and 100%, respectively [14]. The detection rates of NIPT for fetal aneuploidy and CNV > 20 Mb were reported to be 100% [15]. NIPT was an effective method for prenatal screening of fetal CNV ranging from 1 to 129 Mb, with a sensitivity of 84.2% [16]. The findings of this study indicated that NIPT was a reliable method for prenatal screening of fetal pathogenic microdeletion/duplication. However, NIPT cannot detect fetal polymorphism, polyploids, balanced translocations and other fetal structural abnormalities. Therefore, the combination of maternal age, maternal serum screening, fetal ultrasound scanning and NIPT had been recommended for prenatal screening in some studies [17]. Our findings were partially in agreement with this perspective. For pathogenic CNV, the sensitivity of single prenatal diagnosis indication was 0.99 (0.98, 0.99), but the specificity was 0.10 (0.09, 0.11). When prenatal diagnosis indications increased from two to four, the sensitivity was decreased to 0.02 (0.01–0.03) and the specificity increased to 1.00 (1.00–1.00). According to our data, the combination of two screening method was possibly to achieve a maximal summation of sensitivity and specificity. Moreover, each combined screening program had its own advantages and limitations, which require comprehensive consideration by the couples and doctors.

In short, we would recommend that combined at least two kinds of prenatal screening could be used as the efficient program if medical resources for prenatal care are sufficient and the couples are willing to receive the screening.

## Conclusions

Combination of cytogenetic karyotyping and CNV-Seq significantly improves the detection rate of fetal pathogenic chromosome microdeletion/duplication. NIPT was recommended for the screening of pathogenic chromosome microdeletion/duplication, and NIPT combining with other screening methods further improved the screening performance for pathogenic fetal CNV.

## Data Availability

The datasets from the current study are available from the corresponding author upon request.

## Abbreviations

ACMG: American college of medical genetics and genomics
AMP: Association for molecular pathology
Auto A: Autosome aneuploidy
Auto AM: autosome aneuploidy mosaic
B-CNV: benign copy number variations
CAP: College of american pathologists
CMA: Chromosome microarray analysis
CNV: Copy number variation
CNV-Seq: Copy number variation-sequencing;
DS: Down’s syndrome, trisomy 21 syndrome
ES: Edward’s syndrome, trisomy 18 syndrome
FGR: Fetal growth restriction
ISCN: International system for human cytogenetic nomenclature
LB-CNV: Likely benign copy number variations
LP-CNV: Likely pathogenic copy number variations
NF: Nuchal fold
NIPT: Noninvasive prenatal testing
NT: Nuchal translucency
P-CNV: Pathogenic copy number variations
PS: Patau ‘s syndrome, trisomy 13 syndrome
Sex A: abnormal sex chromosome number
Sex AM: abnormal sex chromosome number mosaic
VUS- CNV: copy number variations of uncertain significance
